# Age and sex differences in microvascular responses during reactive hyperaemia

**DOI:** 10.1113/EP091652

**Published:** 2024-03-20

**Authors:** Tom Citherlet, Antoine Raberin, Giorgio Manferdelli, Gustavo R. Mota, Grégoire P. Millet

**Affiliations:** ^1^ Institute of Sport Sciences University of Lausanne Lausanne Switzerland; ^2^ Institute of Health Sciences Federal University of Triangulo Mineiro Uberaba Brazil

**Keywords:** age, microvascular reactivity, sex

## Abstract

Microvascular impairments are typical of several cardiovascular diseases. Near‐infrared spectroscopy (NIRS) combined with a vascular occlusion test provides non‐invasive insights into microvascular responses by monitoring skeletal muscle oxygenation changes during reactive hyperaemia. Despite increasing interest in the effects of sex and ageing on microvascular responses, evidence remains inconsistent. Therefore, the present study aimed to investigate the effects of sex and age on microvascular responsiveness. Twenty‐seven participants (seven young men and seven young women; seven older men and six older women; aged 26 ± 1, 26 ± 4, 67 ± 3 and 69 ± 4 years, respectively) completed a vascular occlusion test consisting of 5 min of arterial occlusion followed by 5 min reperfusion. Oxygenation changes in the vastus lateralis were monitored by near‐infrared spectroscopy. The findings revealed that both women (referring to young and older women) and older participants (referring to both men and women) exhibited lower microvascular responsiveness. Notably, both women and older participants demonstrated reduced desaturation (−38% and −59%, respectively) and reperfusion rates (−24% and −40%, respectively) along with a narrower range of tissue oxygenation (−39% and −39%, respectively) and higher minimal tissue oxygenation levels (+34% and +21%, respectively). Women additionally displayed higher values in resting (+12%) and time‐to‐peak (+15%) tissue oxygenation levels. In conclusion, this study confirmed decreased microvascular responses in women and older individuals. These results emphasize the importance of considering sex and age when studying microvascular responses. Further research is needed to uncover the underlying mechanisms and clinical relevance of these findings, enabling the development of tailored strategies for preserving vascular health in diverse populations.

## Abstract (highlights)

- **What is the central question of this study?**
  How do sex and age influence microvascular responses during post‐occlusive reactive hyperaemia?
- **What is the main finding and its importance?**
  Both women and older individuals showed lower microvascular responsiveness, highlighting the importance of considering sex and age when studying microvascular responses. This may have important implications for understanding vascular health and disease risk across diverse populations.

## 1 INTRODUCTION

The quantification of vascular responses during reactive hyperaemia induced by arterial occlusion is a fundamental assessment of vascular function, both at the macro‐ and at the microvascular level (Rosenberry & Nelson, 2020). Microvascular responses, a critical component of overall cardiovascular health, play a pivotal role in regulating blood flow, oxygen delivery, removal of waste products and nutrient exchange within tissues (Okabe et al., 1990). Therefore, dysfunction in microvascular regulation has been implicated in various pathological conditions such as hypertension (Mitchell et al., 2004), diabetes (López‐Galán et al., 2023) and cardiovascular diseases (Anderson et al., 2011; Huang et al., 2007; Ishibashi et al., 2006). Moreover, impairments at the microvascular level were shown to proceed later with alteration at the conduit artery level (Gutterman et al., 2016). Consequently, assessing microvascular responses is of paramount importance for understanding the underlying mechanisms of these diseases and potentially identifying early markers of their onset.

Doppler ultrasound is the clinical standard method to measure reactive hyperaemia and focus on flow‐mediated dilatation (FMD), a measure of the percentage change in artery diameter serving as a proxy for conduit artery endothelial function. FMD is typically higher in women due to the protective effects of oestrogen and lower in older individuals due to age‐related endothelial alterations. Additionally, the age‐related decline in FMD is less marked in premenopausal women compared to men, but its deterioration accelerates with the reduction in oestrogen levels at the menopausal transition (Green et al., 2016; Raberin et al., 2023).

While FMD provides crucial insights into vascular function at the conduit arteries, near‐infrared spectroscopy (NIRS) has recently emerged for assessing reactive hyperaemia at the microvasculature level (Barstow, 2019). It has proven to be a non‐invasive and reliable method to evaluate microvascular responses (Barstow, 2019; Rosenberry & Nelson, 2020). This technique utilizes the distinct absorption properties of haemoglobin and myoglobin in response to near‐infrared light and provides real‐time information about tissue oxygenation, blood volume and blood flow. It can be used during a vascular occlusion test (NIRS‐VOT).

While most of the studies have focused on conduit arteries, the potential sex and age differences in the microvasculature remain largely unexplored (Molbo et al., 2022). Recent NIRS‐VOT studies have suggested that women have decreased microvascular responses as shown by slower desaturation and reperfusion rates (Fellahi et al., 2014; Keller & Kennedy, 2021; Keller et al., 2023; Rasica et al., 2022; Traylor et al., 2023), higher minimal tissue saturation (Rasica et al., 2022) and lower reperfusion amplitude (Fellahi et al., 2014; Rasica et al., 2022). Recent studies have reported that age also decreases microvascular responses, as demonstrated by lower desaturation rate (Rogers et al., 2023), lower peak tissue saturation (Horiuchi & Okita, 2020; Rogers et al., 2023) and reperfusion amplitude (Rogers et al., 2023). These differences are not always consistent across the studies since no differences were also noted between individuals of different ages (de Oliveira et al., 2019; Iannetta et al., 2019).

The effects of sex and age remain poorly investigated. Thus, this study aimed to investigate whether ageing and sex influence microvascular responses as assessed by NIRS‐VOT. We hypothesized a lower microvascular response in older compared to young adults, and in women compared to men.

## 2 METHODS

### 2.1 Ethics approval

All participants gave their written informed consent. The study was approved by the local ethics committee (CERVD: 2021–02135) and conformed to the standards set by the latest version of the *Declaration of Helsinki* (excluding registration in a database).

### 2.2 Participants

A total of 27 participants took part in the study, comprising seven younger men (age: 26 ± 1 years, height: 186 ± 8 cm, weight: 83 ± 6 kg, body mass index (BMI): 24 ± 1 kg/m^2^), seven younger women (age: 26 ± 4  years, height: 166 ± 5 cm, weight: 60 ± 7 kg, BMI: 21 ± 1 kg/m^2^), seven older men (age: 67 ± 3 years, height: 174 ± 4 cm, weight: 72 ± 6 kg, BMI: 24 ± 2 kg/m^2^), and six older women (age: 69 ± 4 years, height: 163 ± 9 cm, weight: 64 ± 7 kg, BMI: 24 ± 4 kg/m^2^). Participants completed a physical activity questionnaire (Voorrips et al., 1991) to ensure comparable fitness status. The younger group (YG) obtained a mean score of 17 ± 6, which did not significantly differ (*P* = 0.242) from the mean score of the older group (OG) of 14 ± 8.

In the younger group, participants were required to be <30 years and have a body mass index <30 kg/m^2^, while they had to be between 60 and 75 years old and have a body mass index <35 kg/m^2^ in the older group. All the women in the YG were tested in the early follicular phase of the menstrual cycle. All participants were cleared for exercise by a medical doctor and reported no history of chronic diseases.

### 2.3 Experimental protocol

Each participant performed a vascular occlusion test in a seated position on a cycle ergometer. Oxygenation changes in the vastus lateralis muscle were measured concomitantly during a 5‐min vascular occlusion test followed by 5 min of reperfusion. Muscle oxygenation was assessed by a NIRS device (Portamon, Artinis Medical Systems, Elst, The Netherlands). This device featured three dual‐wavelength (760 and 850 nm) light transmitters–channels at a distance of 30, 35 and 40 mm, respectively, from the receiving optode. The NIRS probe was placed longitudinally over the belly of the right vastus lateralis muscle, ∼10 cm above the knee joint, and was firmly secured and shielded from external light using an elastic bandage. The skin underlying the NIRS probe was carefully shaved and cleaned prior to initiating the experiment. To perform the vascular occlusion test, a pneumatic cuff was placed proximally on the right thigh of the participant and connected to a rapid cuff inflator (E20, Hokanson, Bellevue, WA, USA). After a minimum of 2 min with a stable tissue saturation index (TSI) signal, a 5‐min arterial occlusion (>150% of the systolic blood pressure, with a minimum of 250 mmHg) was induced, followed by a 5‐min reperfusion.

**FIGURE 1:**
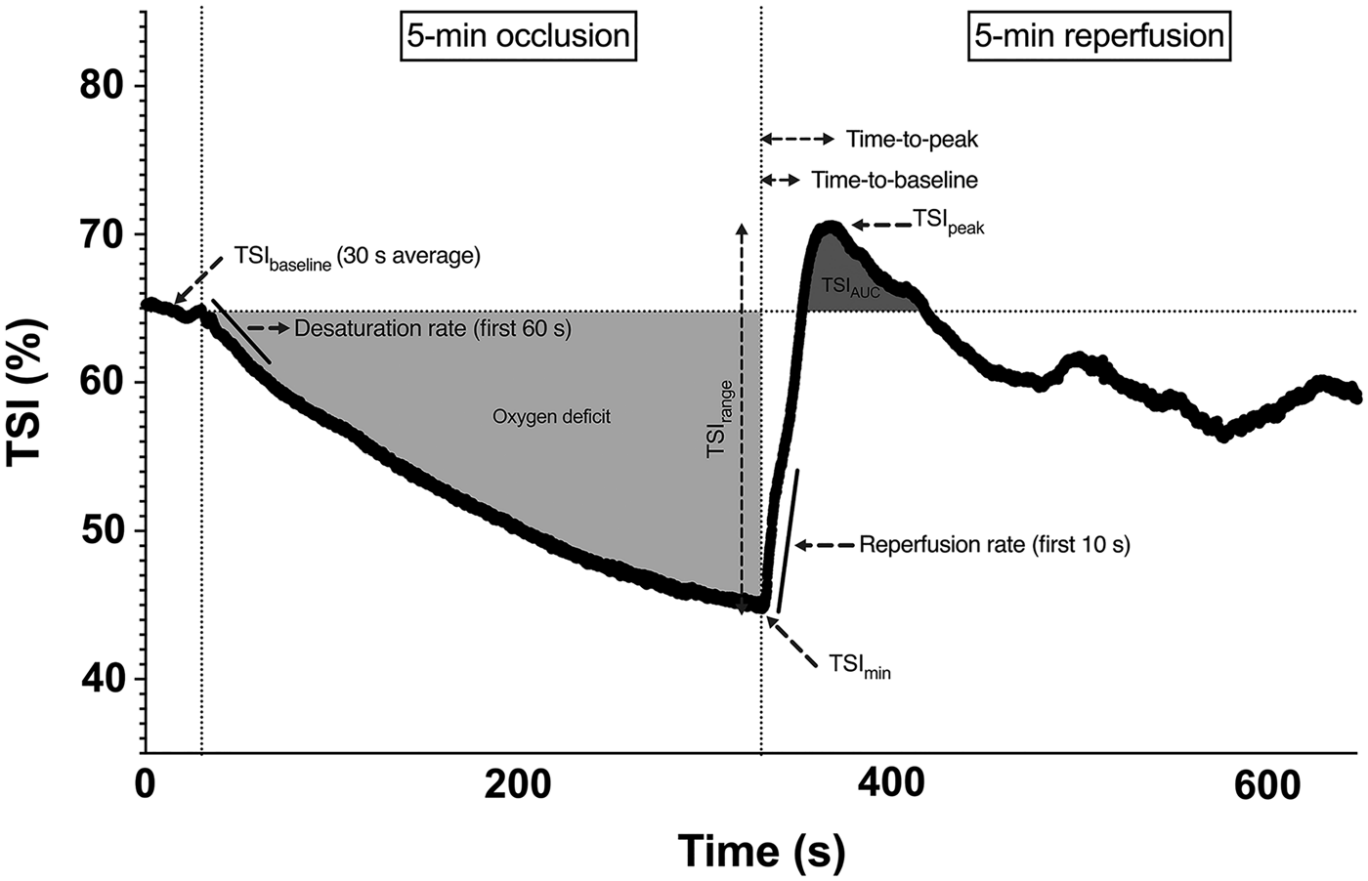
Example of vascular occlusion with the different parameters considered for analysis. TSI_min_, lowest TSI value during the occlusion; TSI_peak_, highest TSI value after the cuff release; TSI_range_, TSI_peak_ − TSI_min_; TSI_AUC_, area under the reperfusion curve above the baseline value until 2 min following the cuff release; oxygen deficit, area above the desaturation curve representing the ischaemic stimulus.

Several parameters were computed as follows and are shown in a standard participant (Figure 1): (a) TSI_baseline_ calculated as the 30‐s average of the TSI signal before occlusion; (b) the desaturation rate as the decline rate of the TSI trace during the 60 s period immediately following cuff inflation; (c) the rate of reperfusion as the upslope of TSI signal during the first 10 s following cuff release; (d) TSI_min_ and TSI_peak_ as the lowest and highest TSI values observed during the occlusion and reperfusion phases, respectively, and TSI_range_ corresponding to their difference; (e) time‐to‐baseline and time‐to‐peak as the times needed for the TSI signal to return to TSI_baseline_ and to reach TSI_peak_, respectively, during reperfusion; (f) TSI_AUC_ as the area under the reperfusion curve above TSI_baseline_ during the first 2 min of reperfusion; and (g) oxygen deficit calculated as the area above the desaturation curve under TSI_baseline_ during the occlusion, which represents the ischaemic stimulus. To account for the individual ischaemic stimulus during the occlusion phase, the rate of reperfusion was also normalized for oxygen deficit (Rosenberry & Nelson, 2020). The desaturation rate was taken as an index of skeletal muscle resting oxidative metabolism (McLay et al., 2016), and the reperfusion rate as an index of microvascular responsiveness (Rogers et al., 2023). Time‐to‐baseline and time‐to‐peak are affected by both the microvascular responses and skeletal muscle oxidative metabolism activity (Soares et al., 2020). These endpoints were integrated into our study as they offer insight into the vascular and metabolic adjustments within the tissues, despite not correlating with FMD (Soares et al., 2020). Data were recorded at a continuous rate of 10 Hz and exported at 5 Hz for analysis.

### 2.4 Statistical analysis

A power analysis performed with GPower indicated that, based on the reperfusion rate reported in a previous study (Rasica et al., 2022), a two‐tailed approach, an α‐level of 0.05, an allocation ratio of 1, and a statistical power of 0.8, a minimum of 16 participants would be required to detect potential sex‐related difference.

A two‐way ANOVA was performed to assess the effects of sex and age on microvascular responses (GraphPad Prism, version 9.5.0; GraphPad Software, Boston, MA, USA). Normality was examined using the Shapiro–Wilk test, and homogeneity of variances was verified with Levene's test. The assumptions of normality and/or homogeneity of variance were not met for four parameters (normalized reperfusion rate, TSI_peak_, time‐to‐peak and TSI_AUC_) and a robust ANOVA was carried out (Walrus in Jamovi version 2.0.0.0). The significance level was set at *P *< 0.05.

## 3 RESULTS

**FIGURE 2:**
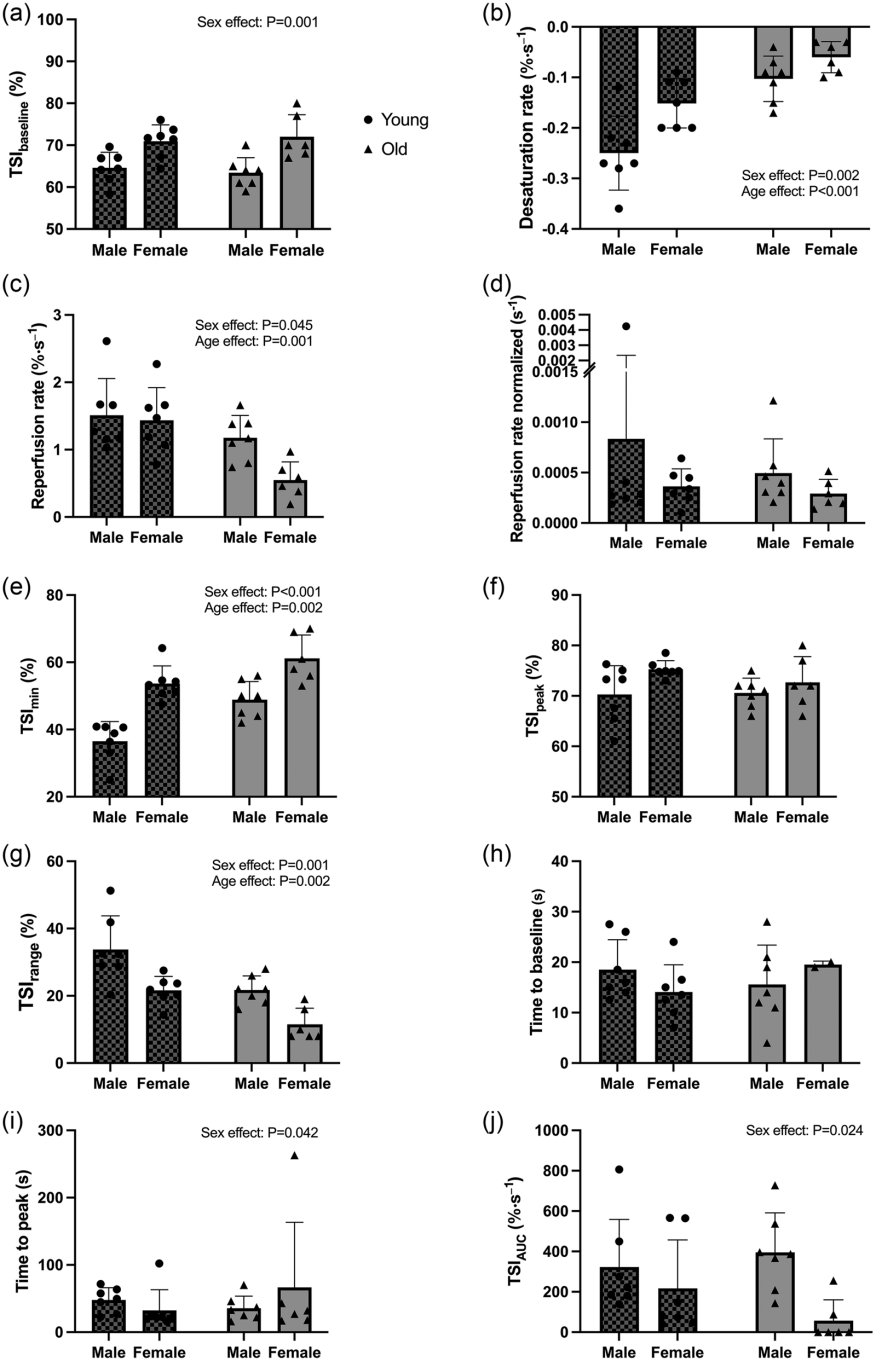
Main tissue oxygenation indexes during vascular occlusion test in the following groups: young men (*n* = 7), young women (*n* = 7), older men (*n* = 7), and older women (*n* = 6). Values are means ± SD. TSI_min_, lowest TSI values during the occlusion; TSI_peak_, highest TSI values after the cuff release; TSI range, TSI_peak_ − TSI_min_; TSI_AUC_, area under the reperfusion curve above the baseline value until 2 min following the cuff release. Reperfusion rate is normalized with respect to the oxygen deficit.

**FIGURE 3:**
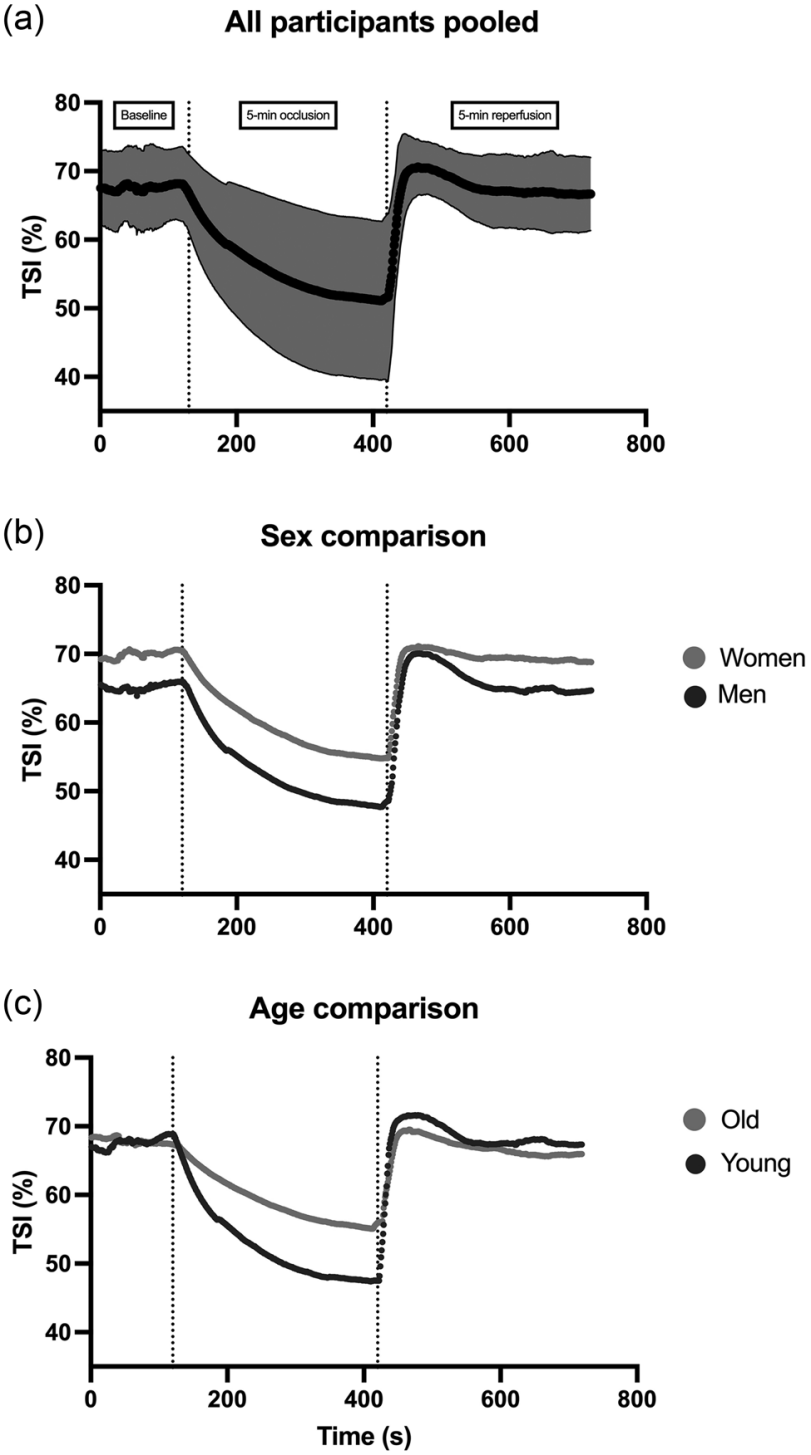
Mean TSI trace of all the participants during the vascular occlusion test. (a) The combined data of all 27 participants. (b) Data for the 14 men and 13 women. (c) Data for the 14 younger and 13 older participants. SD is displayed only in (a) for improved visibility.

Figure 2 shows the calculated average parameters, while Figure 3 displays the average TSI trace for each group.

### 3.1 Sex effect

In women, significantly higher values were found for TSI_baseline_ (+12%), TSI_min_ (+34%) and time‐to‐peak (+15%). Significantly lower values were found for desaturation rate (−38%), reperfusion rate (−24%), TSI_range_ (−39%) and TSI_AUC_ (−60%), but there was no significant difference in TSI_peak_, reperfusion rate normalized and time‐to‐baseline. Among older women, 67% did not reach the baseline after occlusion, whereas this outcome was not observed in older men or young individuals (these cases were excluded from the analysis of time‐to‐baseline and TSI_AUC_). Oxygen deficit was significantly lower in women (3291 ± 1736 vs. 4076 ± 2457% s^−1^, *P* = 0.014).

### 3.2 Age effect

In older participants, significantly lower values were found for desaturation rate (−59%), reperfusion rate (−40%) and TSI_range_ (−39%). A significantly higher value was found for TSI_min_ (+21%), but there was no difference in TSI_baseline_, reperfusion rate normalized, TSI_peak_, time‐to‐baseline, time‐to‐peak and TSI_AUC_. Oxygen deficit was significantly lower in older participants (2409 ± 891 vs. 4895 ± 2282% s^−1^, *P* = 0.001).

## 4 DISCUSSION

This study investigated the independent and combined effects of age and sex on microvascular responses. The main findings were: (a) women exhibited lower microvascular responses expressed by lower desaturation rate, reperfusion rates and TSI_AUC_; (b) older individuals exhibited lower microvascular responses, as shown by lower desaturation and reperfusion rates; (c) normalization of the reperfusion rate to the oxygen deficit abrogated both sex and age differences.

### 4.1 Effect of sex

In agreement with the literature (Fellahi et al., 2014; Keller & Kennedy, 2021; Rasica et al., 2022; Traylor et al., 2023), the present study showed a slower desaturation rate in women. This observation has been attributed to a greater fibre I/fibre II ratio, higher capillary density per unit of muscle and greater mitochondrial respiration (Keller & Kennedy, 2021). A lower strength level has also been proposed as a potential mechanism since normalization for strength was shown to reduce this sex difference in some (Keller & Kennedy, 2021) but not in all (Traylor et al., 2023) studies.

In line with the existing literature (Fellahi et al., 2014; Keller et al., 2023; Rasica et al., 2022; Traylor et al., 2023), a slower reperfusion rate was observed in women. Previous reports normalized reactive hyperaemia measurements against the oxygen deficit (Rosenberry et al., 2019) or normalized the ischaemic stimulus by modulating the occlusion duration (Rosenberry et al., 2018) and found that the disparities between groups observed in the first instance were no longer evident, underscoring that the ischaemic stimulus is a key factor driving the following reperfusion phase. Accordingly, our results showed that the ischaemic stimulus (i.e., the oxygen deficit) was lower in women and that normalizing the reperfusion rate to the oxygen deficit abrogated this difference. Nevertheless, a previous study matched groups for the ischaemic stimulus and did not eliminate the observed sex difference in reperfusion rate (Keller et al., 2023). While the research is not clear yet, the reduced reperfusion rate in women has been ascribed to factors such as diminished microvascular dilatation (Rasica et al., 2022), variations in mitochondrial function, muscle fibre type and/or muscle mass (Keller et al., 2023; Traylor et al., 2023).

Similarly to previous studies (Fellahi et al., 2014; Rasica et al., 2022), women had a smaller TSI_range_ even if we used a different calculation method (TSI_peak_ minus TSI_min_ instead of TSI_baseline_). This is probably explained by a similar TSI_peak_, which is consistent with most existing data (Fellahi et al., 2014; Rasica et al., 2022), but higher TSI_baseline_ and TSI_min_. Strengthening this observation, we also report a smaller TSI_AUC_. These results reinforce the hypothesis of a lower microvascular responsiveness in women.

The higher TSI_baseline_ and TSI_min_ in women can be explained by higher adipose tissue thickness in women as it affects NIRS’ light penetration and leads to higher TSI values (Barstow, 2019; Niemeijer et al., 2017). Previous research confirmed the higher TSI_min_ (Rasica et al., 2022) but did not find a different TSI_baseline_ (Keller et al., 2023; Rasica et al., 2022).

Despite the established link between microvascular dysfunction and macrovascular risks (Gutterman et al., 2016), along with the evidence of greater endothelial function measured by FMD (Holder et al., 2019) and lower incidence of cardiovascular events in women (Iorga et al., 2017), we observed diminished microvascular responses in women.

The disparity in these findings may be explained by the specificities of the assessment techniques. NIRS focuses on microvascular activity, evaluating the response of smaller blood vessels influenced by several factors including, but not limited to, endothelial cell and smooth muscle functions. It also provides insight into skeletal muscle oxygen utilization within tissues, which has been recently recognized as a pivotal component of the hyperaemic response (Rosenberry & Nelson, 2020). In contrast, FMD assesses macrovascular function, measuring large artery endothelial function, that is, influenced by nitric oxide availability. While oestrogens have been demonstrated to upregulate the synthesis of vasodilators such as prostacyclin and nitric oxide, and to attenuate the production of vasoconstrictive substances (Tostes et al., 2003), their effects may predominantly benefit the microcirculation and not necessarily translate to the microcirculation.

### 4.2 Effect of age

In line with the literature (de Oliveira et al., 2019; Horiuchi & Okita, 2020; Rogers et al., 2023), TSI_baseline_ was not impacted by age. We report a lower desaturation rate with age, suggesting a lower muscle oxidative metabolism, in line with some (Rogers et al., 2023) but not all (de Oliveira et al., 2019; Horiuchi & Okita, 2020) previous studies.

Similarily to Rosenberry *et al.* (2018), we found that the reperfusion rate was slower, while others reported it to be identical (de Oliveira et al., 2019; Horiuchi & Okita, 2020; Iannetta et al., 2019; Rogers et al., 2023). Of interest, Rosenberry and Nelson (2020) also found differences in reperfusion rate with age but reported that they were abrogated with the standardization of the oxygen deficit. In accordance, the oxygen deficit was lower in older individuals in the present study, and normalizing the reperfusion rate with the oxygen deficit abrogated the difference, showing that the level of reactive hyperaemia often depends on the magnitude of tissue desaturation.

TSI_peak_ was not different, but TSI_range_ was lower in older individuals, due to a higher TSI_min_. These results remain unclear as it was previously found to be higher (Rosenberry et al., 2018) or identical for TSI_min_ (Horiuchi & Okita, 2020; Rogers et al., 2023), as well as lower (Horiuchi & Okita, 2020; Rogers et al., 2023; Rosenberry et al., 2018) or identical for TSI_peak_ (de Oliveira et al., 2019).

To conclude, the speed of muscle desaturation seems lowered with age suggesting lower muscle oxygen consumption. Age seems to reduce microvascular responsiveness even with normalization of the ischaemic stimulus.

### 4.3 Methodological considerations

Firstly, due to the limited statistical power resulting from our sample size, we were unable to examine the combined influence of sex and age. Secondly, the accuracy of NIRS measurements may be influenced by factors like skin melanin content or adipose tissue thickness. Technical challenges related to ultrasound in this experimentation compromised the measurement of adipose tissue thickness and thereby resulted in the absence of these relevant data. Nevertheless, it is crucial to emphasize that the TSI signal is a relative measurement, representing the ratio of oxygenated haemoglobin to total haemoglobin. Both oxygenated and total haemoglobin are similarly affected by adipose tissue thickness, thus their ratio remains unchanged. Therefore, it may not be necessary to adjust for adipose tissue thickness when utilizing the TSI signal changes (Barstow, 2019). Furthermore, no significant sex differences in the relationship between adipose tissue thickness and NIRS‐derived measurements have been reported in the vastus lateralis (Craig et al., 2017). Finally, it is essential to note that dynamic changes in TSI, such as desaturation and reperfusion rates, should remain independent of adipose tissue thickness, as they are relative measurements rather than absolute ones (Bopp et al., 2014). Consequently, our main outcomes are likely only minimally affected by differences in adipose tissue thickness.

### 4.4 Conclusion

In conclusion, this study demonstrated that women and older individuals exhibit lower microvascular responses, underscoring the importance of considering both sex and age when examining microvascular responses. Normalizing the microvascular responses to the ischaemic stimulus can abrogate differences, which helps in understanding the nature of these differences. Sex and age play pivotal roles in shaping various facets of microvascular responses, which, in turn, hold implications for our understanding of vascular health and disease risk. As we move forward, further studies are required to confirm these findings and unravel the underlying mechanisms driving these age and sex differences. By gaining deeper insights into age‐ and sex‐related variations in microvascular responses, we can develop more precise strategies for promoting and preserving vascular health across diverse populations.

## AUTHOR CONTRIBUTIONS

Conception or design of the work: Tom Citherlet, Antoine Raberin, Giorgio Manferdelli Data acquisition and analysis: Tom Citherlet, Antoine Raberin, Giorgio Manferdelli, Gustavo R. Mota All authors interpreted the data for the work, drafted the work or revised it critically for important intellectual content, approved the final version of the manuscript, and agreed to be accountable for all aspects of the work in ensuring that questions related to the accuracy or integrity of any part of the work are appropriately investigated and resolved. All persons designated as authors qualify for authorship, and all those who qualify for authorship are listed.

## CONFLICT OF INTEREST

The authors declare no conflicts of interest.

## Data Availability

The raw data supporting the conclusions of this article will be made available by the authors, without undue reservation.
